# A topical ointment formulation containing leaves’ powder of *Lawsonia inermis* accelerate excision wound healing in Wistar rats

**DOI:** 10.14202/vetworld.2020.1280-1287

**Published:** 2020-07-07

**Authors:** Kalbaza Ahmed Yassine, Hemida Houari, Benchohra Mokhtar, Amara Karim, Salem Hadjer, Bediaf Imane

**Affiliations:** 1 Department of Veterinary Sciences, Institute of Veterinary and Agronomic Sciences, University of BATNA-1, Algeria; 2 Laboratory of Agro-Biotechnology and Nutrition in Semi-Arid Regions, University of Tiaret, Algeria; 3 Department of Animal Health, Institute of Veterinary Sciences, University of Tiaret, Algeria

**Keywords:** excision, *Lawsonia inermis*, petroleum jelly, Wistar rats, wound healing

## Abstract

**Aim:**

*Lawsonia inermis* (LI), a naturally grown or cultivated shrub in Northeast of Africa and India, has been traditionally used as a strong remedy for several injuries. However, few studies have reported its use as a cicatrizing agent. The aim of this study was to evaluate the effect of daily application of an ointment prepared with LI leaves’ powder on wound healing in Wistar rats.

**Materials and Methods:**

Twenty female Wistar rats were used in this study. Excisional wound model was realized by removing skin from the dorsal part of the neck of each animal. Wounds have been then treated by a daily application of LI ointment prepared by mixing leaves’ powder to petroleum jelly in test group and by simple application of petroleum jelly in control group. Evaluation of wound healing activity was then based on calculating the percentage of wound contraction, period of epithelialization, and wound index every 3 days for a period of 24 days, then, a histological study of the healed excised wound was performed.

**Results:**

Treatment with LI has shown excellent wound healing activity, since it has increased percent of wound contraction, and reduced period of epithelialization and wound index as compared to control (p<0.05). These results have been supported by the histological findings that revealed better epithelialization, dermal differentiation, collagen fiber orientation, and angiogenesis in LI treated rats compared to control (p<0.05).

**Conclusion:**

We can conclude that LI leaves’ can be used as a potential wound healing agent.

## Introduction

A wound is a rupture produced in the skin, which disrupts its cellular and anatomical structures and affects its functionality [1]. After a wound, complex processes and cascade of cellular events are set up to reconstruct the injured part and restore its tensile strength [2]. The wound healing process takes place in three overlapping phases; the hemostasis/inflammatory phase, the proliferation phase, and the ­remodeling phase [3]. The inflammatory phase is characterized by the usual signs of inflammation such as heat, pain, and edema. It is a natural response to the wound, during which the wound’s blood vessels vasoconstrict to form a blood clot. The blood vessels then expand after hemostasis, to allow immune cells, some growth factors, enzymes, and nutrients to reach the wound. During proliferation, a new granulation tissue composed of collagen and an extracellular matrix are formed to reconstruct the wound. This new tissue is then invaded by a new network of blood vessels. Then, the epithelial cells are formed in the wound surface in a process called epithelialization. Finally, after closure of the wound, the phase of remodeling or maturation of collagen occurs [4]. Wound healing is influenced by several factors such as bacterial infection, medications, and wound site location [5]. Other factors also affect wound healing, namely, aseptic conditions, removal of necrotic tissues, approximation of wound edges, and regular application of dressings [6]. The delay in healing can have serious consequences and leads to amputation in complicated cases. Therefore, the search for new agents that can improve the healing process is of great importance and many efforts have been made in this area [7].

The use of traditional medicinal plants for wound healing is based on their antiseptic, astringent, anti-inflammatory, and antibacterial properties [8]. Moreover, it has been reported that medicinal plants contain many substances that enhance wound healing process [6], such as saponins, alkaloids, tannins, steroids, and glycosides [5]. However, it is ­challenging to select a plant based on its activity and careful attention is needed to determine its value [7].

Henna (*Lawsonia* *inermis* [LI]) is a shrub that grows naturally in the tropical and subtropical regions of Africa, Asia, and Australasia. Its dyeing qualities are best when cultivated between 35°C and 45°C in dry conditions [9]. At a temperature below 5°C, the plant dies. Henna is a much-branched, glabrous shrub up to 6 m tall. Its bark is grayish brown, unarmed when young and with branchlets in older plants. Its leaves are opposite, elliptic to broadly lanceolate. They contain a coloring molecule called lawsone. Its pyramidal small white flowers are gathered in clusters and give off a captivating scent. Fruits are small red berries that turn brown when dry [10]. Henna has several medical uses. This plant is used by people because of its antimicrobial and astringent activities. It is also declared as a hypotensive, antihemorrhagic, and sedative agent. However, its main use is cosmetic as a dye for hair and skin [11].

To the best of our knowledge, studies that have reported its use as a wound healing agent are rare [1,11]. The aim of this study was to assess the healing effect of LI leaves’ in the form of an ointment on excisional wounds in Wistar rats.

## Materials and Methods

### Ethical approval

The study was undertaken after approval of experimental protocol by the Ethical Committee of the Institute of Veterinary and Agronomic Sciences – BATNA 1 University – Algeria.

### Study period and location

The study was carried out during the period from October to December 2019. The clinical part of the study was realized at the Institute of Veterinary Sciences at the University of Batna 1, and then the histopathological treatment was finished at the Institute of Veterinary Sciences in University of Tiaret.

### Animals

Twenty healthy female albino Wistar rats (120-140 g) obtained from Pasteur Institute of Algeria were used in this study. Before starting the experiment, animals were housed for 10 days in well-ventilated and temperature-controlled room (25±2°C). They have been maintained in clean rodent cages, where they have received standard rat pellet feed (UAB El-KSEUR BEJAIA – Algeria) and *ad libitum* water in clean rodent bottles. Cleaning of the cages beds and water bottles was carried out daily.

### Plant and ointment preparation

LI leaves were gathered from the region of TIARET in West Algeria. Leaves were dried in the laboratory at an optimal temperature (26±2°C). After complete drying, they were crushed by an electric grinder until a fine powder was obtained. An ointment formulation of 50% was then prepared by adding 50 g of the leaves’ powder to 100 g of petroleum jelly previously heated until being melted 65°C water bath [12]. The ointment formulation was then mixed until a homogenous mixture was obtained.

### Experimental grouping

In this study, we have used two groups of ten animals each: Control (C): treated with simple ointment vehicle (petroleum jelly) and LI group: treated with LI 50% ointment formulation.

### Wound creation

Aseptic conditions were fully respected in all surgical procedures. Wound healing activity of the plant was evaluated on an excision wound model [1]. Before the creation of wounds, animals were anesthetized with an intramuscular injection of ketamine hydrochloride (80 mg/kg) and xylazine (10 mg/kg). After that, the dorsal part of the neck was shaved and the skin to be excised was outlined with a marker. A rectangular excisional wound of 700 m^2^ was then made along the marking using scalpel, sharp scissors, and dissecting forceps. After wound creation, hemostasis was achieved by the application of a sterile gauze soaked in normal saline. Animals have been maintained in their cages and wounds have been left open during all the experiments. LI ointment and petroleum jelly were then applied topically at the wound site since the 2^nd^ day and during all the experiment.

### Measurement of wound area and determination of wound closure percentage

The progressive change in the wound area was monitored on an HD camera on days 0, 3, 6, 9, 12, 15, 18, 21, and 24. Wounds’ pictures have then been uploaded on AutoCAD 2020 (Autodesk, Inc – USA) [13] to calculate the wound area surface. Wound closure percentage was finally calculated using the following formula:


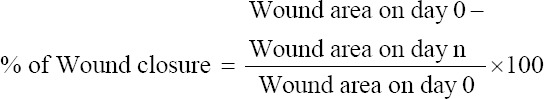


Where n = 3^rd^, 6^th^, 9^th^, 12^th^, 15^th^, 18^th^, 21^st^, and 24^th^ days post-wounding days.

### Determination of epithelialization period

To determine the end point of healing (complete epithelialization), wounds have been observed daily to determine the moment of scab dropping without leaving any wound raw.

### Determination of wound index

Wounds have been observed daily to determine wound index by an arbitrary scoring system [14], as presented in Table-1.

**Table-1:** Wound index scoring system.

Observations	Score
Formation of pus-evidence of necrosis	4
Healing yet not be started but environment is healthy	3
Delayed, but healthy healing	2
Incomplete but healthy healing	1
Complete healing	0

### Histological evaluation of wound healing

The rats were sacrificed at the 24^th^ day by a cardiac injection of propofol. Tissues were excised from the wound site of each animal and have been separately stored in 10% formalin solution. After undergoing the usual dehydration, clarification and paraffin impregnation, simples have been cut to 5 μm sections with a rotary microtome, deparaffinized, mounted on glass slides and stained with hematoxylin and eosin. We could not add Masson Trichrome staining in this study because this coloration is not available in our university due to budget restrictions. Histological assessment was then performed by observation of the glass slides under the microscope.

Histological evaluation was performed using a modified scoring system based on the previous models adapted by Sultana *et al*. [15] and Abramov *et al*. [16] (Table-2). Evaluation of wound healing was based on the assessment of epithelialization, epidermal differentiation, amount of granulation tissue, inflammation, collagen fiber orientation, and neovascularization. Total histological score was finally obtained by adding scores of different assessed parameters.

**Table-2:** Modified histological scoring system.

Score	Epithelialization	Differentiation	Amount of granulation tissue	Inflammation	Collagen fiber orientation	Neovascularization
1	Absente	Absent	Profound	Severe	Vertical	<5/HPF
2	Moderate	Present	Moderate	Moderate	Mixed	6-10/HPF
3	Marked	/	Absente	Weak	Horizontal	>10/HPF

HPF=High power field

### Statistical analysis

The results of wound area measurement, wound closure percentage, period of epithelialization, wound index, and histological scores of wound healing were calculated and expressed as mean±standard deviation (SD). Obtained data were subjected to one-way analysis of variance to determine the significant difference between control and treatment group, using Minitab computer software (version 19). Dunnett’s *t*-test was used to analyze the intergroup significance and p<0.05 was considered as statistically significant.

## Results

### Wound area and wound closure percentage

**Figure-1:**
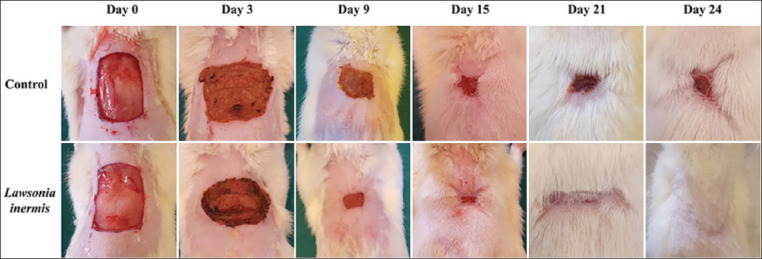
Gross appearance of excision wound healing during the 24-day study period (Wound scar areas have been shaved in some animals to allow better viewing).

Wound area (mm^2^) measured in all animals on days 0, 3, 6, 9, 12, 15, 18, 21, and 24 is summarized in Table-3. Treatment with LI has shown excellent wound healing activity during the entire experiment period. On the 3^rd^ day post-wounding, the percentage of wound closure was already 39.81% in LI group compared to 13.99% only in the control group (p<0.01). This has continued during the following days to reach 78.28% on the 9^th^ day compared to 60.17% in the control group (p<0.01). The percentage of contraction of the wound in rats treated with LI has ranged then from 89.23% to 99.85% in the period of the 12^th^ to 15^th^ days compared to 75.79-96.37% in the control group on the same period (p<0.01). After 24 days, animals treated with LI have presented a completely healed wound, as shown in Figure-1.

**Table-3:** Measurements of wound areas over a period of 24 days showing the percent of wound contraction in both groups.

Group	0 day	3^rd^ day	6^th^ day	9^th^ day	12^th^ day	15^th^ day	18^th^ day	21^st^ day	24^th^ day	POE
C	626.78±41.77 0	607±61.79 13.99±4.52	317.09±30.29 54.80±5.44	276.36±43.52 60.17±10.21	166.98±67.02 75.79±11.50	97.00±45.51 85.97±7.81	50.31±23.99 92.82±3.76	30.56±18.33 95.63±2.83	25.21±15.59 96.37±2.28	23.70±0.90
LI	619.09±66.54 0	373.96±65.53 39.81±5.92	207.97±37.63 66.24±5.94	131.05±52.66 78.28±9.90	65.08±34.27 89.23±6.01	21.34±11.90 96.44±2.17	5.49±2.08 99.06±0.90	3.41±2.00 99.40±0.60	0.88±0.50 99.85±0.16	20.10±1.20
p-value	0.764	0.000	0.000	0.001	0.004	0.001	0.000	0.001	0.000	0.000

C=Control, LI=*Lawsonia inermis*, POE=Period of epithelialization

### Period of epithelialization

Mean±SD of period of epithelialization is summarized in Table-3. The shedding of the scab has lasted for an average of 20 days without leaving any residual wound scar in the group treated with LI ointment. However, in control group, since the wounds were still unhealed after 24 days, the scab was still present.

### Wound index

Mean±SD of wound indices is represented in Table-4. Rats treated with LI, compared to control group, have shown better wound indices throughout the experiment (p<0.01). On 3^rd^ day, wound index recorded in LI group was 2.50±0.70 whereas it was 3.90±0.31 in control. On day 24, it reached 0.50±0.32 in LI group, which was very significantly lower (p<0.01) than that recorded in control group (1.60±0.51).

**Table-4:** Mean±SD of wound index in both groups.

Group	3^rd^ day	6^th^ day	9^th^ day	12^th^ day	15^th^ day	18^th^ day	21^st^ day	24^th^ day
C	3.90±0.31	3.60±0.69	3.50±0.85	2.80±1.31	2.30±1.25	2.40±1.17	1.70±0.48	1.60±0.51
LI	2.50±0.70	2.60±0.84	2.00±1.41	1.40±0.51	1.40±0.51	1.10±0.31	0.70±0.48	0.50±0.32
p-value	0.000	0.010	0.010	0.006	0.050	0003	0.000	0.000

C=Control, LI=*Lawsonia inermis*

### Histological score of wound healing

**Figure-2:**
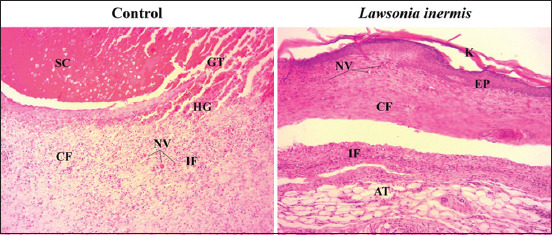
Histological sections of healed excised wound showing marked epithelialization and epidermal differentiation, horizontal collagen fiber orientation, more than 10 blood vessel per high power field in LI group and moderate epithelialization, mixed collagen fiber orientation, 6-10 blood vessel per high power field in control group; hematoxylin and eosin; 100×; AT=Adipose tissue, CF=Collagen fiber, EP=Epithelialization, GT=Granulation tissue, IF=Inflammatory cells infiltrate, K=Keratin, NV=Neovascularization, SC=Scab.

**Figure-3:**
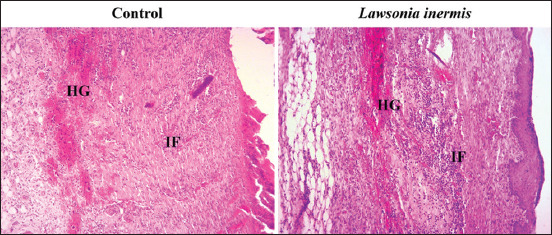
Histological sections of healed excised wound showing presence of profuse hemorrhage and inflammatory cells infiltration in both groups; hematoxylin and eosin; 100×; IF=Inflammatory cells infiltrate, HG=Hemorrhage.

Mean±SD of histopathological scores of wound healing is summarized in Table-5. Animals treated with LI have shown better histopathological scores compared to those of control group (p<0.05). In fact, LI group has shown a marked epithelialization and epidermal differentiation, a horizontal orientation of collagen fiber and more than 10 blood vessels per high power field compared to a moderate epithelialization, a mixed collagen fiber orientation and 6-10 blood vessels per high power field (Figure-2). We have also noticed that the application of LI ointment has resulted in a marked inflammatory cells infiltration compared to control group. However, no difference has been found in terms of granulation tissue amount between both groups. Finally, we have noticed the presence of a profuse hemorrhage in all histological sections (Figure-3).

**Table-5:** Mean SD of histopathological scores of both groups.

Score	Epithelialization	Differentiation	Amount of granulation tissue	Inflammation	Collagen fiber orientation	Neovascularization	Total histopathological score
C	2.33±0.87	1.67±0.50	2.22±097	1.56±0.73	2.56±0.53	2.00±0.00	12.33±3.08
LI	3.00±0.00	2.00±0.00	2.75±0.46	2.25±0.46	3.00±0.00	3.00±0.00	16.00±0.76
p-value	0.047	0.080	0.182	0.035	0.031	0.000	0.005

C=Control, LI=*Lawsonia inermis*

## Discussion

Wounds are commonly encountered in animals and humans. If these wounds are not treated timely, the injured tissues can suffer from serious complications such as infections, and chronic inflammation [17]. Chronic wounds cause functional impairment and high morbidity if they are not treated seriously. It is estimated that around 6 million people worldwide suffer from chronic injuries, which is a significant number and therefore causes a considerable increase in treatment costs [18,19].

Medicinal plants have been widely used as an alternative to allopathic medicines in the treatment of several diseases [20]. It has been reported that an improvement in the healing rate and a decrease in pain, discomfort and scarring for the patient are observed while using certain herbal remedies [21,22].

LI is a plant native of North Africa and South West Asia. Widely known as Henna, its ground and dry leaves have been used for several years in cosmetics. People use Henna for staining hands, hair dye, and in folk-medicine as a prophylactic against skin diseases [23]. Therefore, LI is considered a safe cosmetic material [24] and well regarded by the cosmetic industry [25]. Roots and leaves are the most commonly used parts of plants in wound healing studies [26]. The present study was performed to evaluate the healing effect of LI leaves’ after their daily application in the form of ointment on excisional wound in rats.

In our study, animals treated with LI ointment have shown better wound contraction than those of the control group since 40% of the wound area was already covered in the LI group after only 3 days, compared to only 14% in control group. This wound contraction has continued during the following days and after 15 days the wound was almost completely covered. This increase in the rate of wound contraction can be explained by a shortening of the inflammatory phase due to antimicrobial activity of the plant and stimulation of inflammatory cells. It is also possible that this improvement is the result of an amelioration of proliferation phase linked to a mitogenic activity of the plant stimulating the proliferation of fibroblasts and their differentiation into myofibroblasts with effective contractile activity. These effects on wound healing process have resulted in a shortening of the epithelialization period and after 20 days the wounds were completely covered with new skin. The results of our study are similar to those reported in the few studies that evaluated the effect of this plant on wound healing [1,11,27]. The wound contraction is the result of the contractile activity of myofibroblasts and the movement of the epithelial cells formed during the epithelialization phase [28,29]. This process involves complex interactions of cells, the extracellular matrix, and cytokines. Plants are thought to improve wound healing by stimulating fibroblasts which therefore migrate from the edge to the wound site, proliferate, and produce collagen [30].

A close observation of healed wound histological sections confirms our *in vivo* results as it has revealed that regeneration was much more rapid in the treated group compared to control group. In fact, histological sections have shown an enhanced epithelialization and epidermal differentiation, marked infiltration of inflammatory cells, increased blood vessel formation, an enhanced proliferation and organization of fibroblasts as a result of LI application. Increased inflammatory cells infiltration observed in LI treated rats compared with control may be due to a chemotactic effect of the plant, which resulted in an attraction of inflammatory cells toward the wound site. Moreover, increased cellular proliferation and differentiation confirm the mitogenic activity of the plant, which have enhanced the healing process. We can also confirm that the plant has enhanced cellular proliferation, granulation tissue formation, and epithelialization since dermal and epidermal regeneration was better in treated rats. Furthermore, marked angiogenesis observed in LI treated rats proves that the plant stimulates the angiogenesis process. Our results are in agreement with those reported by the only report that has performed a histopathological study of wound healing after application of this plant [1].

The therapeutic value of derived plants compounds lies in their production of certain physiological actions on the organism [31]. The main compounds involved in wound healing are alkaloids, flavonoids, tannins, terpenoids, saponins, and phenolics [32]. Although our study has shown that LI significantly improves the rate of wound contraction and the epithelialization period in rats, it is difficult to correlate these beneficial effects with a specific component of this plant, as long as this effect can be attributed to a single component or result from a combined activity of several active metabolites.

Many studies have described the chemical composition of LI dried leaves [33-35]. A recent study has reported that LI is composed of phenolic compounds (coumarins, flavonoids, tannins, naphthalenes, naphthoquinones, xanthones, lignans, alkylphenones, etc.), terpenes, steroids, alkaloids, miscellaneous, and some minerals [36].

Among these compounds, coumarins, ­flavonoids, tannins, and alkaloids are the most involved in wound healing process. In fact, it has been reported that coumarins could ameliorate wound healing due to their antioxidative activity and edema protective function [37,38]. Moreover, flavonoids enhance wound healing by their astringent and antibacterial activities [39-41], their prevention of cell necrosis, improvement of angiogenesis [42], inhibition of prostaglandin synthesis [43], and modulation of cytokines expression during the inflammation phase [44]. Tannins have also been reported to improve wound healing by improving the regeneration and organization of new tissue through their astringent and antibacterial activities, their antioxidant power, and their anti-inflammatory and antifungal effects [45-47]. Tannins contribute also to several mechanisms such as free radical chelation, which promotes improved wound contraction and angiogenesis [48], and stimulates the proliferation of fibroblasts and keratinocytes [49]. In addition, tannins contribute to rapid crust formation by precipitating proteins in damaged tissue. This also reduces the permeability of the capillaries in the wound by reducing edema and tissue exudation [49,50]. Finally, alkaloids could facilitate wound healing by blocking the metabolic pathway of arachidonic acid [51].

## Conclusion

We can conclude from this study that the topical application of an ointment prepared from LI leaves’ powder improved wound healing in an excisional model. The LI ointment used in our study showed better results because the wound contraction and the epithelialization period were better than those observed in control group. These findings are asserted by the histological founding after 24 days, which showed an almost healed skin with good epithelialization and differentiation as well as good angiogenesis. This study can be taken as a benchmark for further investigations which will aim to isolate the various components of the plant and determine their exact effects on wound healing.

## Authors’ Contributions

KAY and AK designed the study and performed the surgical procedures. BM and HH realized the histological study. SH and BI helped in the clinical follow-up. All authors read and approved the final manuscript.
